# Micro-computed tomography permits enhanced visualization of mycangia across development and between sexes in *Euwallacea* ambrosia beetles

**DOI:** 10.1371/journal.pone.0236653

**Published:** 2020-09-21

**Authors:** Ellie Spahr, Matt T. Kasson, Teiya Kijimoto

**Affiliations:** Division of Plant and Soil Sciences, West Virginia University, Morgantown, West Virginia, United States of America; University College London, UNITED KINGDOM

## Abstract

Symbiosis can facilitate the development of specialized organs in the host body to maintain relationships with beneficial microorganisms. To understand the developmental and genetic mechanisms by which such organs develop, it is critical to first investigate the morphology and developmental timing of these structures during the onset of host development. We utilized micro-computed tomography (μCT) to describe the morphology and development of mycangia, a specialized organ, in the Asian ambrosia beetle species *Euwallacea validus* which maintains a mutualistic relationship with the Ascomycete fungus, *Fusarium oligoseptatum*. We scanned animals in larval, pupal and adult life stages and identified that mycangia develop during the late pupal stage. Here we reconcile preliminary evidence and provide additional morphological data for a second paired set of structures, including the superior, medial mycangia and an inferior, lateral pair of pouch-like structures, in both late-stage pupae and adult female beetles. Furthermore, we report the possible development of rudimentary, or partially developed pairs of medial mycangia in adult male beetles which has never been reported for any male Xyleborini. Our results illustrate the validity of μCT in observing soft tissues and the complex nature of mycangia morphology and development.

## Introduction

Symbiotic relationships can affect the development of the host body. Non-obligate two-partner systems have allowed for detailed examinations of how symbiotic partners may direct development in one another. Recent work in the *Vibrio-Euprymna* squid symbiosis has examined the role the bacterial symbiont *V*. *fischeri* plays in directing and maintaining “mature” morphology in the light organ of the squid [1]. Persistent colonization of the bacterial symbiont is required to efficiently regress surface epithelia and maintain the mature interior organ cell types [1]. Another prominent model for symbiont-directed host morphogenesis can also be found in the Rhizobia-legume symbiosis (as reviewed by Gage 2004) [2]. Presence of the microbial symbiont triggers differentiation within the legume host root to begin the root nodulation process [3]. The legume responds to Rhizobia bacteria by forming primary nodules and modifying root hair structure to allow Rhizobia into the root interior. Once the relationship is established, both nodules and bacteria will differentiate and reach a mature symbiotic state. Bacteria have contributed to both animal and plant symbioses and as such are prominent models for studying development in mutualistic partnerships. Fungi have also contributed to a number of important symbioses, yet their role in shaping host development remains unclear.

Fungus farming, perhaps the best-known example of fungus-mediated symbiosis, has evolved in multiple insect lineages as either a sole nutrition source or to supplement dietary needs [4]. Ecology and evolution of fungus farming has been studied extensively within Hymenoptera (ants), Blattodea (termites), and Coleoptera (beetles) [4, 5]. In ants and termites, mycophagy has evolved only once and radiated spectacularly leading to the diverse extant clades that exist today [6, 7]. In contrast to ants and termites, beetles have evolved nutritional symbioses with fungi multiple times throughout the Coleopteran order [6, 8].

Ambrosia beetles represent two sub-families of weevils (Curclionidae: Scolytinae, Platypodinae) that maintain obligate nutritional mutualisms. In the ambrosia symbiosis, obligate nutritional symbioses have evolved in parallel with an estimated 11 independent origins of fungus farming. Each relationship has coincided with an independent origin of specialized structures (mycangia) to carry symbiotic propagules within or on the insect body [5, 8, 9]. These structures develop internally or externally across the head, thorax, and abdomen and may vary from shallow pits in the exoskeleton to complex sacs or tubular structures [9]. Some mycangia have shown evidence of glandular cells, but in-depth categorization of potential secretory function has not been thoroughly investigated across mycangia [10, 11]. Additionally, in-depth anatomical studies of single beetle species across life stages or sex have never been formally investigated.

*Euwallacea* ambrosia beetles have received significant attention over the past decade because of the damage they inflict on a variety of trees within invaded habitats around the world including Israel, Australia, Mexico, the United States, and, most recently South Africa [12–15]. Native to Asia, these beetles have caused serious damage to avocado production worldwide and impacted numerous woody plant hosts in both cultivated and forest environments [16]. One species of particular interest, *Euwallacea validus*, introduced from Asia to the Eastern United States in the late 1900s [17], possesses paired oral mycangia and vectors two fungal species; the primary nutritional symbiont *Fusarium oligoseptatum* and *Raffaela subfusca*, a secondary fungus recovered consistently from female heads from introduced populations across the eastern U.S., but whose role remains unclear [13, 18].

Xyleborine beetles—including *Euwallacea* spp.*—*reproduce via haplodiploid sib-mating [19]. As in typical haplodiploid reproductive systems, diploid females selectively fertilize eggs to generate female offspring whereas unfertilized haploid eggs give rise to male progeny that will then mate with their siblings within the gallery tunnel [20]. Female beetles play major roles in mediating the symbiosis with *F*. *oligoseptatum* by dispersing them between host trees whereas males are reduced in size and flightless; function within the gallery is relegated to reproduction and possibly fungal maintenance [20].

To date, paired oral mycangia have been described in adult female Xyleborini using both destructive and non-destructive sectioning techniques. Investigations with microtome cross-sectioning (a destructive method), such as the report of *Xyleborus glabratus* by Fraedrich et al. (2008), *Xyleborus affinis* by Hulcr and Cognato (2010) and *Ambrosiodmus lecontei* by Li et al. (2015) have revealed basic structural organization and confirmed the presence of fungal propagules [21–23]. More detailed structural investigations of mycangia in Xyleborini and closely related taxa (Premnobiina) have built upon microtome methodology using emerging non-destructive imaging techniques. The mycangia of *Ambrosiodmus lecontei*, *A*. *minor*, *Ambrosophilus atratus*, *Premnobius cavipennis*, and *Euwallacea interjectu*s were observed across multiple methods (microtome, LAT scan, and μCT) [24, 25].

For *Euwallacea* ambrosia beetles, there is considerable, albeit fragmented evidence regarding mycangia organization and structure (Table 1). Fernando (1960) illustrated a single pair of superior medial oral mycangia for *E*. *perbrevis* (*= X*. *fornicatus*; tea shot hole borer in Sri Lanka) in both transverse and sagittal orientation [26]. Work by Nakashima (1982) used microtome cross–sectioning to reveal a pair of mycangia at the bases of the mandibles of *E*. *validus* (*= X*. *validus*), although the image was considerably distorted [27]. Reports from Goto (1998) described for the first time a new type of mycangia in *E*. *validus*, *E*. *interjectus*, and a third unresolved *E*. sp. with a pair of inferior pouches located below and lateral to the known paired superior medial oral mycangia, however, no visual evidence (microtome sections or illustrations) was available to support these conclusions [28]. Kasson et al. (2013) [13] used microtome sectioning to confirm the presence of a superior pair of oral mycangia in *E*. *validus*. Freeman et al. (2016) [29] used *Fusarium euwallaceae* expressing GFP to visualize mycangia and confirm *F*. *euwallaceae* occupies a single pair of superior medial oral mycangia. Li et al. (2018) also described a single superior medial mycangia in *E*. *interjectus* using LAT scans [25]. However, the *E*. *interjectus* mycangia described by Jiang et al. (2019) [30] using μCT was observed laterally at a lower plane in the head than presented by Li et al. (2018) [25]. Taken together, these findings reveal that enhanced resolution is needed to reconcile differences in both location and number of paired oral pouches. Furthermore, these previous imaging studies of *Euwallacea* focused exclusively on adult females, which are known to harbor these specialized fungal pouches, thereby limiting our understanding of development across life stage and beetle sex.

**Table 1:** Summary of past research on structure of oral mycangia for *Euwallacea* ambrosia beetles.

Euwallacea sp.	Specific location within head	Evidence	Orientation(s)	Reference
*E*. *fornicatus*	Superior medial	GFP staining		Freeman et al. 2016 [29]
*E*. *interjectus*	Inferior lateral	MicroCT	transverse & sagittal	Jiang et al. 2019 [30]
*E*. *interjectus*	Superior medial	LATscan	transverse	Li et al. 2018 [25]
*E*. *perbrevis*	Superior medial	Illustration	transverse & sagittal	Fernando 1960 [26]
*E*. *sp*.	Superior medial and inferior lateral	Descriptive	N/A	Goto 1998 [28]
*E*. *validus*	Superior medial	Microtome section	indeterminable	Nakashima 1982 [27]
*E*. *validus*	Superior medial	Microtome section	transverse	Kasson et al. 2013 [13]

Here, by integrating two imaging techniques (traditional microtome cross sectioning and μCT) of the entire head, our goal is to enrich the knowledge on mycangial development across life stages of beetles as well as between sexes. Particularly, we focused on examining the developmental timing as well as possible sex-specific development of mycangia in *E*. *validus*. Such studies are also important across the Xyleborini because; 1) previous studies tended to focus on confirmation of a single pair of mycangia providing only a partial or single-plane transverse section of the adult female heads possibly overlooking the presence of secondary structures; and 2) several *Euwallaea* spp. have yielded more than one fungus with similar abundances from their mycangia [13, 31] raising the possibility of co-cultivation in single mycangia or specialized structures adapted for each symbiont [32].

## Materials and methods

### Rearing protocol

Rearing methods were adapted from Cooperband et al. (2016) [33] to account for regional host-preference (*Ailanthus altissima*) of our target ambrosia beetle species, *Euwallacea validus*. *Ailanthus altissima* limbs were felled and collected from the Evansdale campus of West Virginia University in Morgantown, West Virginia. Limbs were cut into 2.5cm long cross sections and dried at room temperature for several weeks. Dried limb material was milled down to 1 mm trapezoidal sawdust particle size for beetle rearing media using a Fritsch Power cutting mill pulverisette 25 (Fritsch GmbH, Germany). Sterilized sawdust agar as described by Cooperband et al. (2016) [33] was prepared in 50mL Falcon tubes.

Beetles were retrieved from infested *Ailanthus altissima* stems in the West Virginia University forest during late spring (June). The stems were cut into 0.6m sections and split longitudinally into quarter sections to reveal gallery tunnels. Beetles of all life stages were collected from the galleries by mechanical force and retrieved using sterile water and paint brushes or soft-grip forceps. Life stages were stored separately. Beetles were then transferred to non-inoculated media and provided “starter” tunnels along the outside of the tube. 5 adult female beetles and up to an additional 3 males were introduced per tube of media and housed in an incubator at 26°C.

### Animal staging

**Fig 1:**
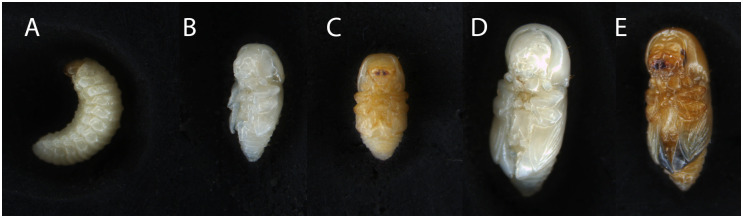
External features of the ambrosia beetle *Euwallacea validus*; A) larva, B) early male pupa, C) late male pupa, D) early female pupa, and E) late female pupa. “Late” and “early” stage designations were made based on coloration of head capsules and elytra. Animals close to eclosion appear darker throughout the entire pupal body.

Pupae were staged and selected based on coloration on head capsule and elytra (Fig 1). Adult females were staged by body coloration (Fig 4); color relates to degree of exoskeleton sclerotization and estimates post-molting adult maturity.

### Micro-computed tomography

*Euwallacea validus* were fixed in 70% ethanol at room temperature at least overnight. Insects were then stained overnight in 5% Lugol solution (I_3_K), modified from Metscher et al. (2009) [34] for iodine in potassium iodide stains.

Scanning was conducted using the Skyscan 1272 μCT (Bruker). Samples were scanned in ethanol in stand-mounted 200 μL pipette tips. Scanning parameters were unified for all samples (2x2 binning at 4.3 μm pixel size, no filter. X-ray source set at 50 kW and 200 μA). Scan data was compiled using NRecon (version 1.7.1.0) and cropped in Dataviewer (version 1.5.3.4). Digital cross-sections and videos were captured from the 3D model in CTVox (version 3.3). Detailed captured movie is available as a supplemental movie (https://doi.org/10.6084/m9.figshare.12221981.v1).

### Microtome cross-sectioning

Samples were fixed in 2% PFA overnight and soaked in phenol for 6 days before paraffin embedding and cross-sectioning (as modified from Li et al. 2015) [23]. Prior to paraffin embedding, insects were re-fixed with formaldehyde then dehydrated in an ethanol gradient series. Samples were stored until sectioning. Transverse sections were taken using a Microm HM 325 rotary microtome (Walldorf, Germany) and slides hand-stained in Harris hematoxylin and eosin-phloxine. Slides were imaged using a Nikon Eclipse E600 compound microscope (Nikon Instruments, Melville, NewYork) and Nikon Digital Sight DS-Ri1 high-resolution microscope camera through Nikon NIS-Elements BR 3.2 imaging software.

## Results

### Detailed description of adult female mycangia

**Fig 2:**
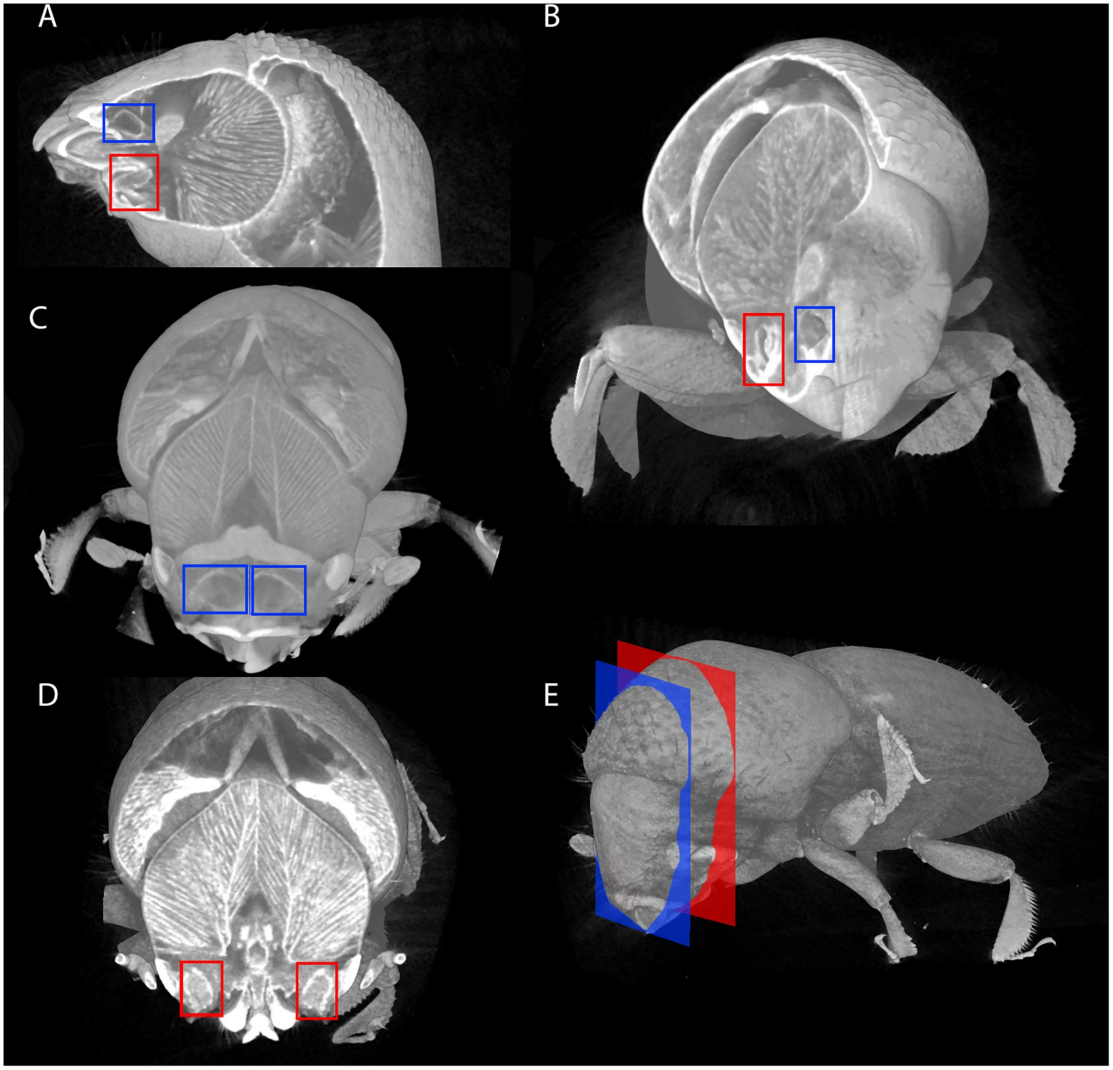
Micro-computed tomography (μCT) digital cross sections of mycangia and inferior paired pouch-like structures in adult female *E*. *validus*. Blue boxes denote superior, medial mycangia (C) while red boxes denote the inferior, lateral structure (D). Plane of visualization for each paired structure (C,D) denoted by corresponding color (E).

**Fig 3:**
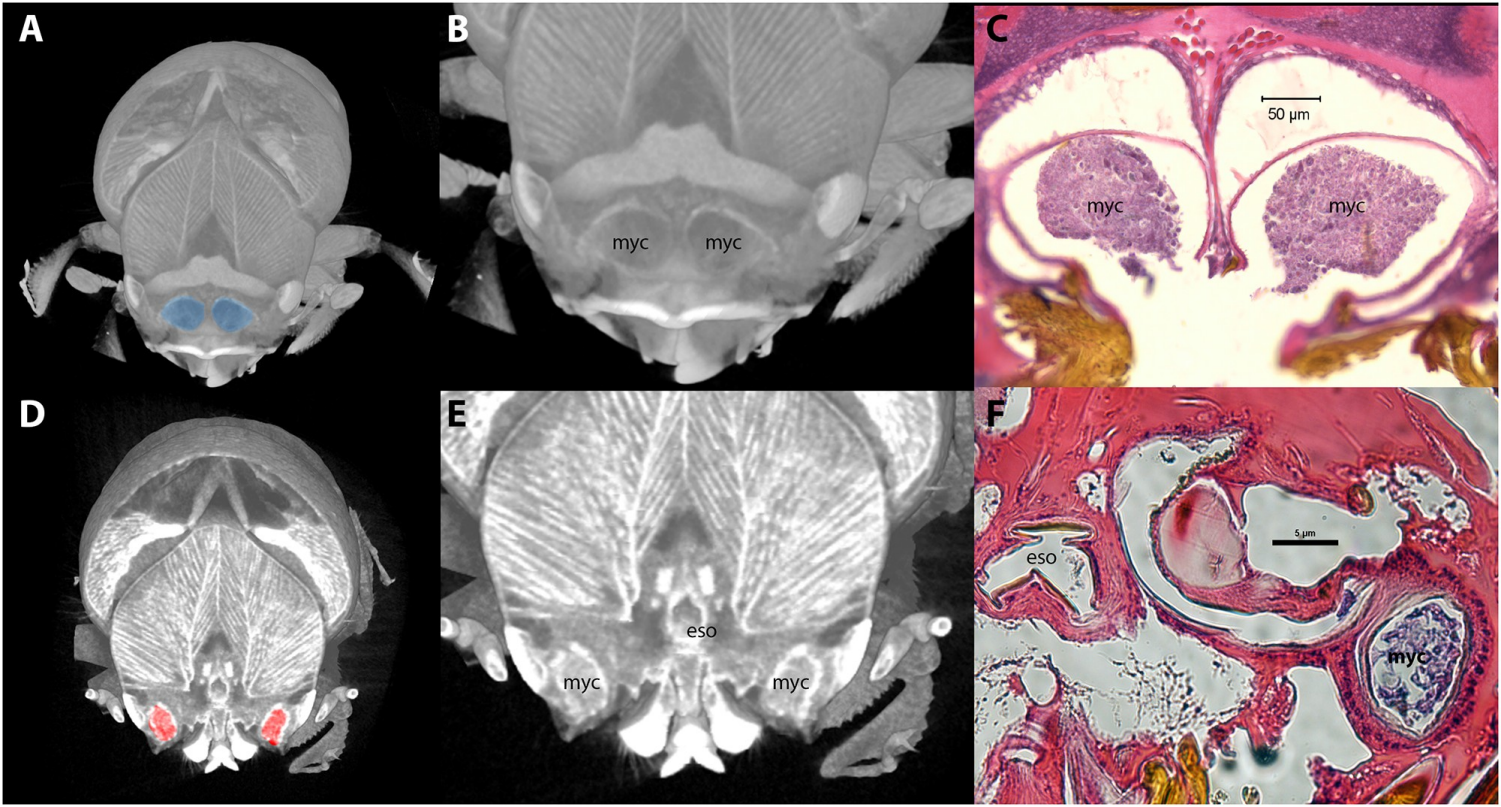
Micro-computed tomography (μCT) and microtome sections for mycangia (A-C) and inferior lateral (D-F) pouch-like structures. Abbreviations “eso” represents the esophagus, “myc” denotes mycangia.

Adult female beetles show two pairs of oral pouches (in all five sampled adult female insects). The first set is organized superomedial to the mandibles in the (rostral) cephalic tissue (Fig 2A, 2B and 2C). The second pair are organized laterally, inferior to the rear of the mandibles, beside each compound eye (Fig 2A, 2B and 2D, also see supplemental movie; https://doi.org/10.6084/m9.figshare.12221981.v1). Microtome cross sectioning revealed interior colonization of both medial superior pockets (Fig 3C) and on the lateral side of the head near the eye (Fig 3F) as equivalent to the inferior pouch-like structures in our μCT images. Columnar cells line the primary membrane of the lateral pouch-like structures (S1B and S1C Fig). Duct-like structures (S1A, S1B and S1E Fig) are present next to the chamber but we did not observe physical connection between these features among the sections we generated. Microtome sections further indicated that beetles develop small spines and hair-like structures within the duct (S1A, S1B, S1D and S1E Fig). Similar columnar cells lining the lateral pouches along with peripheral duct-like and hair-like structures can be seen in the lateral mycangia of closely related beetle genera as well as more distantly related tribes (S2 Fig) [24, 35].

### Mycangial development across life stages in female

**Fig 4:**
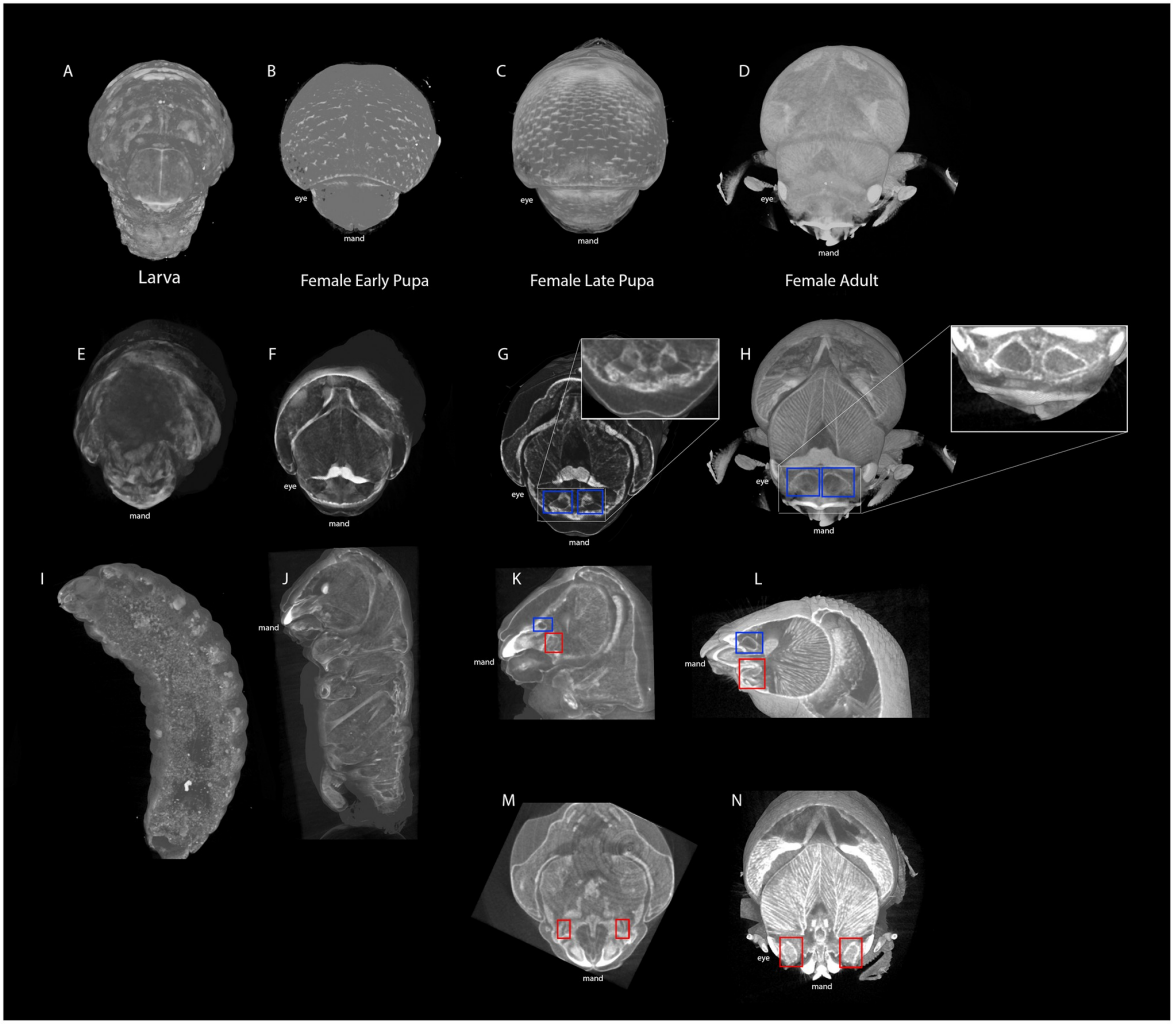
μCT digital cross sections across female *Euwallaca validus* life stages. Blue boxes denote superior, medial mycangia while red boxes denote the inferior, lateral pouches. A-D) External view of the superior transverse cross-sections (E-H). I-L) Sagittal cross-sections reveal superior mycangia and inferior pouches in late pupa (K) and adult (L) female beetles. M, N) Inferior transverse cross-section illustrates paired inferior pouches in late pupa (M) and adult (N) female beetles.

We observed mycangia in different life stages to estimate the developmental timing. As shown in Fig 4E and 4F, we did not observe clear images of mycangia during larval and early pupal stages, however we were able to detect the development of both superior mycangia and inferior structures in the late pupal stage of females. In the early pupal stage, we observed a paired structure with weak contrast in an equivalent space where the superior mycangia develop in late pupae and adults (Fig 4F), suggesting mycangia start developing after pupation.

### Sexual dimorphism of mycangial development

**Fig 5:**
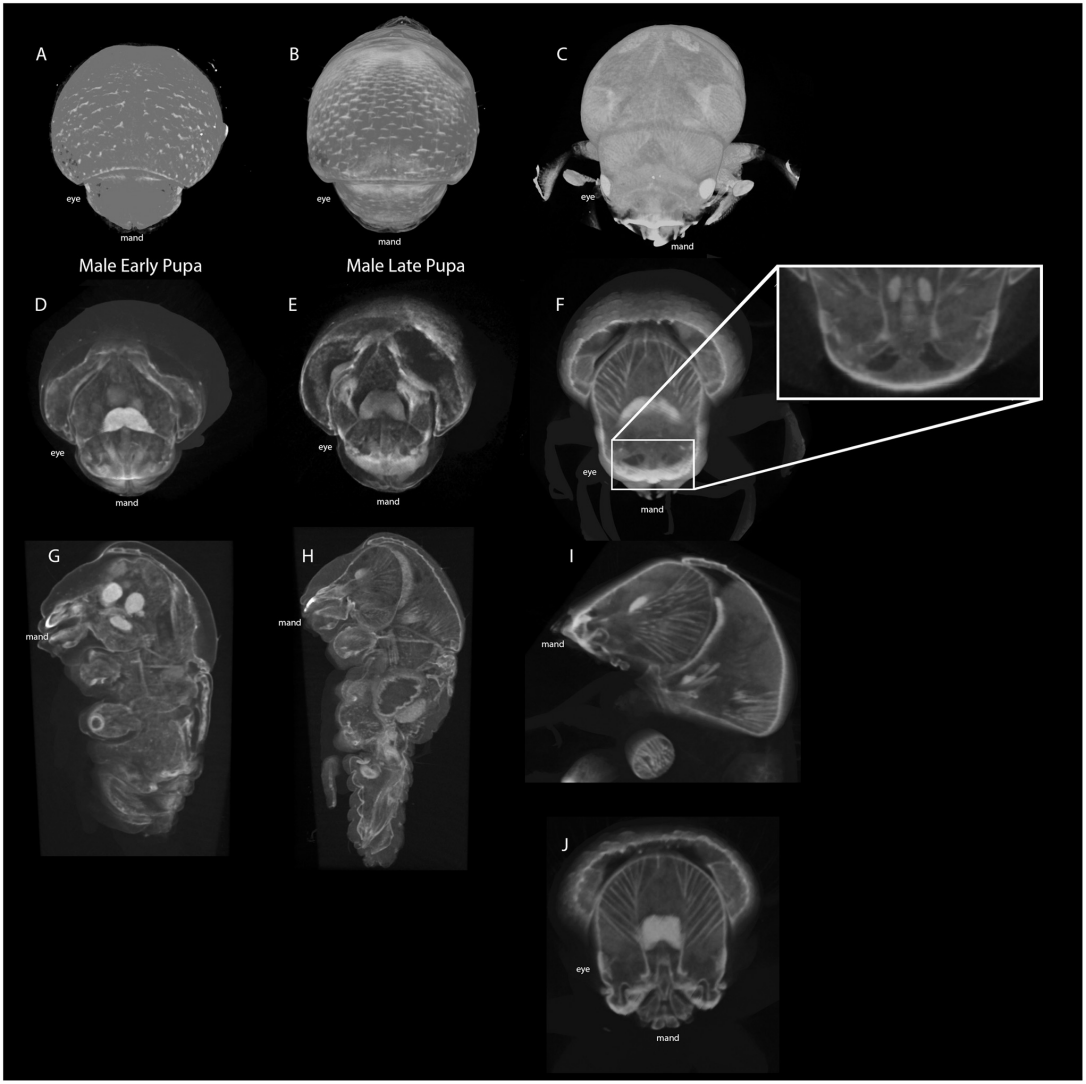
μCT digital cross sections across male *Euwallaca validus* life stages. A-C) External view from which the primary transverse sections (D-F) originate, respectively. G-I) Sagittal sections of male insects. J) Transverse section of adult male head, where lateral pouches were observed in adult females.

We scanned males in equivalent life stages as we did in females. Scanned images suggest that males may harbor mycangia-like spaces (referred to as protomycangia hereafter) in an equivalent position where females develop mycangia (Fig 5F, 5I and 5J). The superior protomycangia do not show a defined boundary whereas membranous boundaries of female mycangia were clearly visible in late pupae and adults (Fig 4G and 4H). The superior protomycangia become observable during late pupal stage in a similar form as observed in the early female pupal stage (compare Figs 4F and 5E). Of 9 adult males scanned to verify this result, 7 showed clear image of the same structure.

## Discussion

Our μCT digital sectioning along with microtome cross-sectioning confirmed the developmental timing of mycangia; they appear to develop during pupal stage in female beetles. In previous studies, structural analyses have focused on describing mycangial structure in the adult female [11, 23, 25, 30, 36]. Here we were able to supplement our knowledge by adding a new piece of information; developmental timing.

Oral mycangial structure has been studied by using beetles from three genera; *Euwallacea*, *Ambrosiodmus*, and *Premnobius*, where organization of mycangia within the head varies between species. In *Euwallacea interjectus* and *Ambrosiodmus* species as published by Li et al. 2015 and 2018, [23, 25] oral paired mycangia are located together in front of the esophagus and directly behind the mandible, homologous to the superior pair of mycangia we observed here. Li et al. (2018) [25] shows *Premnobius* mycangia organized at a lower plane of cross section on either side of the head behind the eyes, reminiscent of the inferior pair of pouches in our observation. Jiang et al. 2019 [30] shows morphological evidence in *E*. *interjectus* which denotes mycangia in a lateral, inferior organization as present in *Premnobius*. If both pairs exist in *Euwallacea* species (as observed separately by Li et al. 2015 and 2018 and Jiang et al. 2019 in *E*. *interjectus*), these membrane-bound structures may be linked, either physically or in function [23, 25, 30]. Indeed, the presence of two sets of pouches may be the “dual paired” mycangia described by Goto for *Euwallacea*, though no image-based evidence is available to corroborate (Goto 1998) [28]. The inferior structures observed here corroborate the independent descriptions of laterally organized structures, providing morphological evidence in a single insect body for pouches in both superior and inferior positions in the head.

Our microtome cross-sectioning results complement the μCT images, further reinforcing the idea that oral mycangia in *E*. *validus* may be more complex than a single pair of oral sacs. Organization within the head suggests that these inferior membrane-bound structures may represent lateral, inferior mycangia as they are positioned between the eye and esophagus, rather than paired in the front of the head (Fig 3F). Isolation of fungal propagules from these pouch-like structures would further support this notion.

The duct-like structure adjacent to of the inferior pouch-like structures of *E*. *validus* contains small hair-like protrusions and spines, both of which were not observed in μCT scanning images. These hair-like structures whose biological function is yet to be elucidated are also notable in microtome sections of *Ambrosiophilus atratus* [35], *Premnobius cavipennis* [24], and, to a lesser extent, *Ambrosiodmus lecontei* [23].

A recent study by Mayers et al. (2020) [32] has described both prothoracic and oral mycangia from female *Xyloterinus politus*, each of which contains a unique fungal symbiont including a *Raffaelea* sp. in the oral mycangium. If spatially separate structures harbor distinct symbionts in *X*. *politus*, colonization of *F*. *oligoseptatum* in only medial *E*. *interjectus* pouches (as observed by Freeman et al. 2016) [29] does not preclude functional colonization of lateral structures. While microtome and/or μCT data has been traditionally sufficient for describing mycangia, the “dual paired” organization obscures the ability to confirm symbionts from head dissection and culture-based methodology. Further work is needed to examine specific constituents and functional contributions of each structure in *Euwallacea*.

Detection of mycangial development during pupal stage facilitates the progress of research on mycangia development in at least two ways: 1) we now know in which stage at the latest we should start exploring the genetic underpinnings of mycangial development and 2) the direct physical contact with their symbiotic fungi may not be required to induce mycangial development, similar to what has been shown for mesonotal mycangia in *Xylosandrus* ambrosia beetles (Li et al. 2019) [37].

As mentioned above, females are responsible for dispersion of fungal propagules between tree hosts and establishing the gallery system with *F*. *oligoseptatum* within the tree. Males are haploid, flightless, and have reduced wing size, mandibles, and body size [20, 21]. In natural habitats, males are relied upon for reproduction and possibly gallery maintenance [20]. Indeed, scan data suggests reduced musculature in reconstructed μCT models of male heads (compare Figs 4H and 5F). This decreased muscle density and reduced mandibular size may reflect their behavioral niche within the symbiosis; reduced musculature can suggest a similar reduction in chewing strength and thus an inability to perform other roles such as building galleries.

Careful observation of the compiled 3D model has revealed dual paired gaps in the male beetle head reminiscent of mycangial pouches. While they do not appear to be distinctly membrane-bound as in female *E*. *validus* (Fig 4 - female and Fig 5 - male), the position in the head seems to be equivalent to female mycangia. This structure, which has not been previously observed by other techniques (microtome cross-sectioning or head dissection), may be a rudimentary tissue of mycangia. The male mycangia-like space (protomycangia) may be reduced in size, similar to mandibles and wings, by a function of ploidy. Male mycangia are known from other ambrosia beetle tribes including the Xyloterini [32], but to our knowledge this represents the first reported male mycangia-like structures in the Xyleborini confirmed with imaging.

Previous morphological and culture-based studies failed to recover meaningful numbers of fungal propagules or reconcile the mycangial structure in adult male *Euwallacea* beetles [13]. Since male *Euwallacea* is not known to vector the nutritional symbiont, this structure may be non-functional. Together with the data provided by our structural investigation, this supports the idea that mycangial development may have ceased prematurely in males. While the current resolution provided by scan data has not been fully conclusive, it has captured structural patterning where microtome sectioning may not be suitable. This illustrates the benefit to conducting multiple methods of morphological confirmation.

In summary, the potential development of protomycangia in males suggests that although the development of mycangia may take place in both sexes, the developmental timing is regulated differently between males and females, resulting in male protomycangia not functioning as in the mycangia in female beetles. More specifically, the development of protomycangia may start later during development than female counterparts, therefore it ceases before maturation (Fig 5). In many beetle species, males develop enormous structures such as horns, which requires longer developmental timing than females that do not develop such traits [38]. In *E*. *validus*, females are the larger sex in body size and therefore may be able to complete the development of mycangia. Detailed life stage study of both sexes (i.e. comparison of time until sexual maturation) would further evaluate the latter notion.

Due to the numerous independent origins of mycangia, development of premandibular mycangia may not be observed in both sexes outside the genus *Euwallacea*. However, we propose that structural development in males should be widely evaluated using multiple techniques to determine whether this is conserved among broader taxonomic groups. A better understanding of when and how mycangia develop between and across sexes would further assist our understanding in mycangial evolution and development.

### Concluding remarks

The results we obtained suggest mycangia, a specialized organ to maintain symbiosis with fungi, appear to be quite complex in *Euwallaca validus* and may include a secondary set of pouches that requires further investigation. Furthermore, our results suggest that mycangia in *E*. *validus* begin developing during the pupal stage. The development of equivalent structures in males (protomycangia) is previously unreported in this species and may cease before maturation. This expanded view of anatomy and development may help reveal new complexity in mycangia within *Euwallacea* and further the Xyleborine tribe. We recognize the increasing importance of integrating imaging technologies such as μCT scanning and more traditional sectioning techniques to observe the morphology of these soft organs effectively.

## Supporting information

S1 Fig Microtome cross-sections of *E*. *validus* A) late female pupa and B-E) adult female. Detail views show C) fungal propagules in the inferior, lateral pouches, D) dorsal duct structure, and E) flanking duct with hair-like structures. Orientation of microtome sections denoted in upper right of 1A; D (dorsal), V (ventral), M (medial), L (lateral). “Myc” (A, B) represents mycangia.
(TIF)

S2 Fig Microtome cross-sections of *Euwallacea validus* (A,B), *Ambrosiophilus atratus* (C,D), and *Premnobius cavipennis* (E,F). Arrows present in detail images (B,D,F) denote a conserved border cell type observed between laterally-arranged mycangia.
(TIF)
