# Supplementary figures and images for: Micro-computed tomography permits enhanced visualization of mycangia across development and between sexes in Euwallacea ambrosia beetles
Source: PLoS One. 2020 Sep 21;15(9):e0236653. doi: 10.1371/journal.pone.0236653 (PMC7505430; doi:10.1371/journal.pone.0236653)

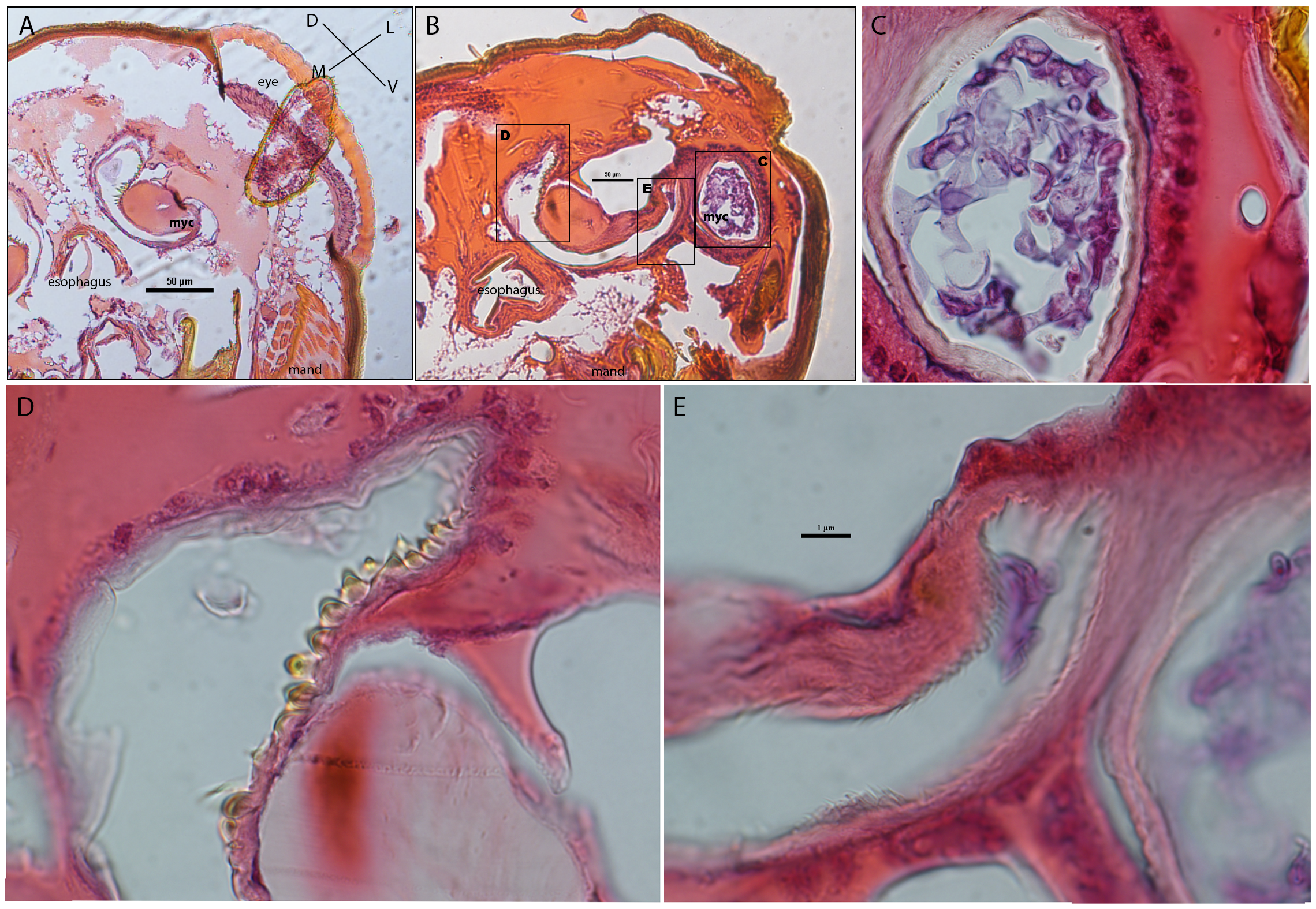

Supplement: S1 Fig — Detail views show C) fungal propagules in the inferior, lateral pouches, D) dorsal duct structure, and E) flanking duct with hair-like structures. Orientation of microtome sections denoted in upper right of 1A; D (dorsal), V (ventral), M (medial), L (lateral). “Myc” (A, B) represents mycangia. (TIF) [file pone.0236653.s001.tif]

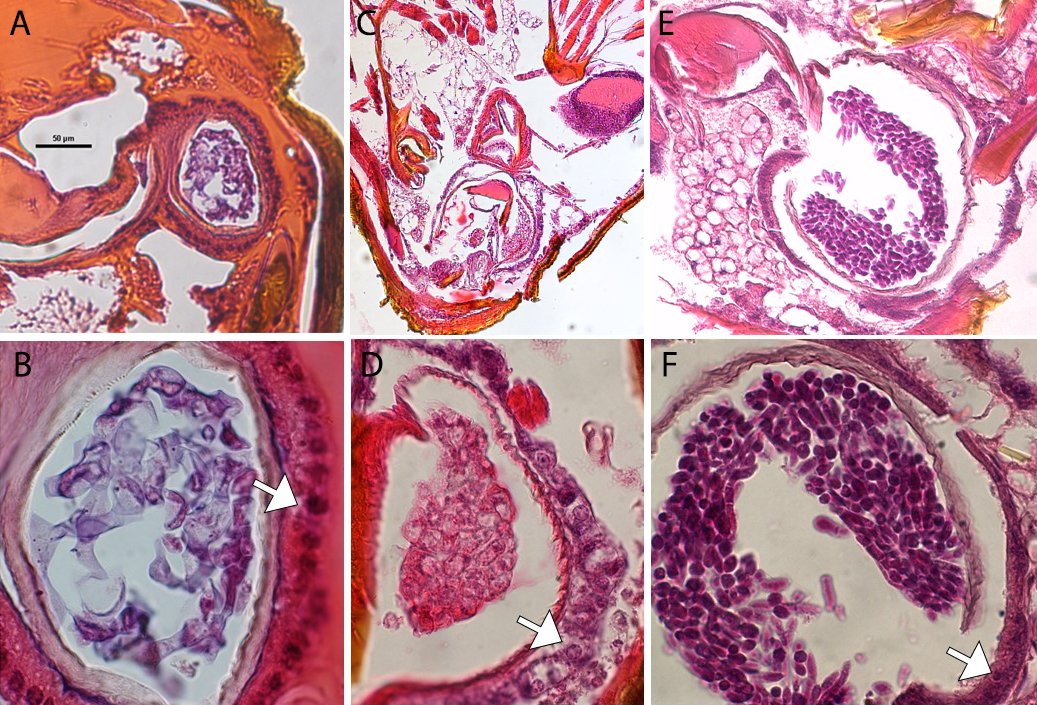

Supplement: S2 Fig — Arrows present in detail images (B,D,F) denote a conserved border cell type observed between laterally-arranged mycangia. (TIF) [file pone.0236653.s002.tif]
